# Juvenile Breast Hypertrophy: A Successful Breast Reduction of 14.9% Body Weight without Recurrence in a 5-Year Follow-Up

**DOI:** 10.1155/2017/3491012

**Published:** 2017-01-31

**Authors:** Akmal Hisham, Marzida Abd Latib, Normala Basiron

**Affiliations:** ^1^Department of Plastic and Reconstructive Surgery, Hospital Kuala Lumpur, Jalan Pahang, 50586 Kuala Lumpur, Malaysia; ^2^Reconstructive Sciences Unit, School of Medical Sciences, Universiti Sains Malaysia, 16150 Kubang Kerian, Kelantan, Malaysia; ^3^Department of Plastic and Reconstructive Surgery, Faculty of Medicine, Universiti Teknologi MARA (UiTM), Sungai Buloh Campus, Selangor, Malaysia

## Abstract

Juvenile hypertrophy of the breast (JHB) is a rare and relentless disease affecting women in the peripubertal period. We present a 13-year-old girl with massive bilateral JHB, successfully treated with a breast reduction and free nipple graft technique. A total of 7300 grams of breast tissue had been removed, accounting for 14.9% of the patient's total body weight. Prophylactic hormonal therapy was not commenced. During the 5-year follow-up period, there was no recurrence and the patient remains satisfied with the aesthetic outcome. A recent meta-analysis study indicates that subcutaneous mastectomy is associated with reduced risk of recurrence, but it is more deforming and the aesthetic result is inferior to a reduction mammaplasty. In patients treated with the latter technique, some evidence exists suggesting that the use of a free nipple graft is associated with a less frequent risk of recurrence than a pedicle technique. This present case is unique as it demonstrates the clinical course of this patient at a considerably longer follow-up period than most reported studies. We adhered to the limited available evidence and highlight the long-term reliability of breast reduction with free nipple grafting as the first line surgical option in JHB, eliminating the need for repeated surgeries.

## 1. Introduction

Juvenile hypertrophy of the breast (JHB) is a benign condition where atypical, alarmingly rapid, and continued breast growth occurs during puberty. It often follows a 6-month period of extreme breast enlargement, superseded by a longer period of slower but sustained breast growth [1]. There are a myriad of terms describing this entity in the literature including virginal hypertrophy, juvenile gigantomastia, and juvenile macromastia [2, 3].

It is a relatively rare condition. Neinstein reviewed 15 publications regarding breast lesions in adolescent spanning a period of nearly 40 years and reported that JHB accounts for only 2% of all breast lesions in this group of patients [4]. In 2011, Hoppe et al. in their meta-analysis of case reports identified 65 reported cases between 1910 and 2009 [3]. Our own literature search, to the best of our knowledge, yielded additional nine cases from 2010 till date [5–11].

The most challenging aspect in the management of JHB is the difficulty in effecting a definitive treatment. The relentless course of this disease and the refractory nature to surgery is well documented [1–3]. Surgical treatment with reduction mammaplasty is ideal but will almost invariably result in recurrence, necessitating secondary reduction or mastectomy.

We report a 13-year-old girl with massive bilateral juvenile breast hypertrophy successfully treated with a breast reduction and free nipple graft technique.

## 2. Case Report

A 13-year-old girl was referred to plastic surgery because of progressive, massive, bilateral breast enlargement for a period of 1 year, plaguing her with severe back and neck discomfort, incapacitating her from school and social activities, and causing her social embarrassment. She attained menarche at 12 years old. Her past medical and family history was unremarkable and she was not receiving any medication.

**Figure 1:**
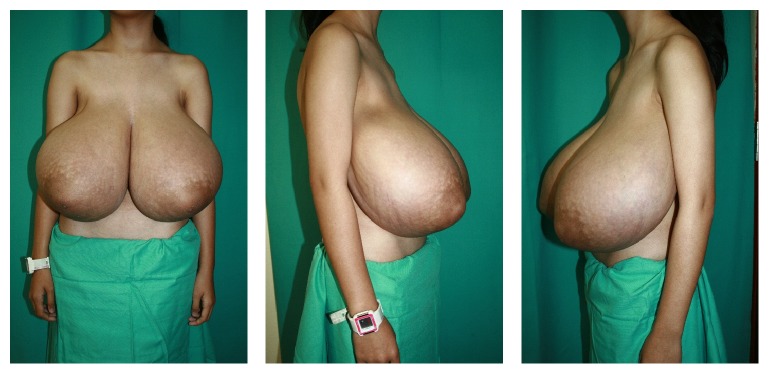
Preoperative anterior and lateral view. The breast measurement was as follows: suprasternal notch-to-nipple distance was 36 cm bilaterally, nipple-to-IMF distance was 26 cm bilaterally, and nipple-to-nipple distance was 21 cm.

On examination, she was a slim girl with a normal BMI of 21.3 kg/m^2^ (weight 49 kg and height 1.50 m). The breast was symmetrical, pendulous, and disproportionately enlarged with widened areolas. Shoulder grooving was present. There was no intertrigo at the inframammary fold (IMF) and the skin quality was good. Palpation revealed a uniformly firm texture without discrete masses. The breast measurements were as follows: suprasternal notch-to-nipple distance was 36 cm bilaterally, nipple-to-IMF distance was 26 cm bilaterally, and nipple-to-nipple distance was 25 cm (Figure 1).

Hormonal levels of luteinizing hormone, follicle-stimulating hormone, and serum oestradiol were within normal limits. Ultrasound of the breast showed interstitial oedema. Magnetic resonance imaging of the breast and ultrasound of the pelvis did not reveal any abnormality.

**Figure 2:**
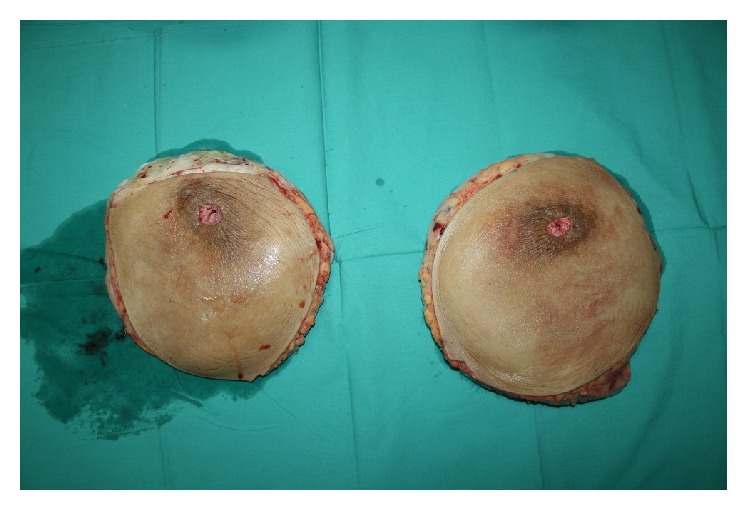
Resected breast tissue. Total amount of 7300 grams of tissue was removed (right breast = 3500 grams; left breast = 3800 grams).

A standard Wise-pattern skin resection was designed and bilateral breast reduction was performed using the free nipple graft technique. A total of 7300 grams of tissue had been resected, accounting for 14.9% of patient total body weight (Figure 2). The resultant defect was closed in an inverted-T scar fashioned closure. No intraoperative blood transfusion was needed. Postoperative period was uneventful and the patient was discharged on day 7 after the operation.

**Figure 3:**
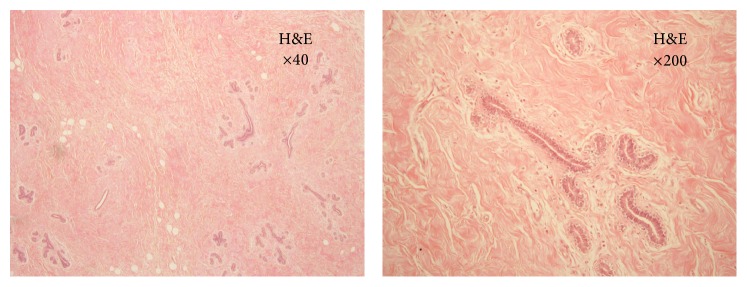
Histological appearance of the left breast in haematoxylin and eosin (H&E) stain. An increase in the interlobular stroma, composed of abundant collagen and fat, was seen. Duct was lined by the normal two-tier epithelium without atypia or hyperplasia. The right breast showed similar appearance.

Histological examination showed an increase in interlobular stroma, composing of abundant collagen and fat. There was perilobular fibrosis in certain areas. Duct was lined by the normal two-tier epithelium without atypia or hyperplasia (Figure 3). The overall clinicohistopathological findings were in keeping with the diagnosis of JHB.

**Figure 4:**
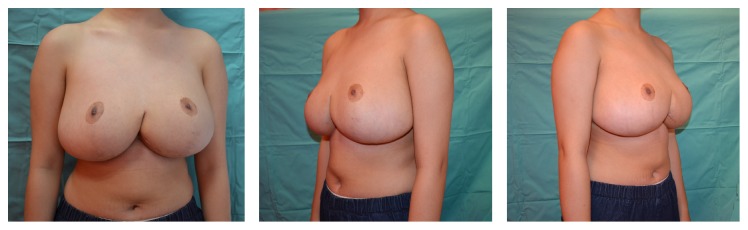
Postoperative views at a 5-year follow-up. No hypertrophic recurrence was observed. Note that the position of the NAC is slightly higher on the left and the breast demonstrates a boxy shape and bottomed-out appearance.

Decision was made not to commence any prophylactic hormonal therapy following a consultation with the paediatric endocrinologist. At a 5-year follow-up, there was no sign of hypertrophic recurrence. However, we noted that the NAC position was higher on the left and the breast demonstrated a boxy shape and a bottomed-out appearance at this stage (Figure 4). Secondary revision was offered but it was declined. She remains asymptomatic and satisfied with the aesthetic outcome.

## 3. Discussion

The exact underlying aetiology for JHB has not been fully elucidated, but several theories have been proposed. The popular theories include end-organ hypersensitivity to normal levels of circulating oestrogen, [12] increased oestrogen or progesterone receptor expression, imbalance of endogenous hormone production, and excessive local oestrogen production [1, 13]. Hereditary and autoimmune causes have also been described, [14, 15] but in most cases the condition is sporadic.

More recently, genetic basis for this disease has also been postulated involving the PTEN (phosphatase and tensin homologue) tumour-suppressing gene. In 2002, Li et al. on a murine model discovered that mutation and deletion of the PTEN gene has been linked to precocious lobuloalveolar development, excessive ductal branching, delayed involution, reduced apoptosis, and mammary epithelial hyperproliferation [16]. However, clinical correlation is still unclear. Two case reports that performed the PTEN gene mutation analysis on their pathologic samples were found to be negative [10, 17]. Our patient had neither the family history nor association with any autoimmune diseases. PTEN gene mutation analysis was not routinely performed in our center.

Clinical features of JHB are similar to those of the adult gigantomastia, albeit the psychological and social sequelae of the gigantomastia are more pronounced in this population of adolescent women [18]. In addition to the mechanical symptoms of back and neck pain, the main complaints of our patient were social embarrassment and avoidance of social activities as a result of her enormous breast size.

Laboratory testing for endocrinology profile, specifically oestradiol, progesterone, LH, FSH, and prolactin, is common practice but is not routinely indicated as it invariably does not reveal any abnormalities [13]. Breast imaging is of limited value owing to the dense breast tissue but should be pursued to rule out tumours [13, 19]. Our patient has a normal endocrinology profile whilst breast sonography and MRI scan excluded other occult breast pathologies.

In most cases, the clinical diagnosis of JHB is so singular and striking that consideration of other breast pathologies is academic [19]. The differential diagnosis of JHB includes giant fibroadenomas, phyllodes tumour, and malignant tumour such as lymphoma and sarcomas. According to Neinstein, the prevalence of these conditions in adolescents with breast lesions was 1%, 0.4%, and 0.9%, respectively [4].

Treatment modalities in JHB involve the following four strategies: (1) surgical management, (2) medical therapy administered either preoperatively or (3) postoperatively, and (4) medical therapy alone [13].

The surgical management options are mastectomy with implant reconstruction and breast reduction (reduction mammoplasty) either as a pedicle-based technique or with a free nipple graft. Hoppe et al. reported a significant relationship (*p* < 0.01) and an odds ratio of 7.0, for the likelihood of recurrence using a reduction mammoplasty as compared with a mastectomy. This finding indicates that mastectomy offers the most definitive treatment for JHB [3]. However, the extent of the procedure, the possible psychological consequences, and the lifelong risk of implant reconstruction limit its use in the adolescent women.

A more common and well-accepted sequence of treatments consists of breast reduction surgery as the first line option, followed by mastectomy with implant reconstruction in the event of recurrence. In patients treated with breast reduction surgery, Fiumara et al. reported some statistical evidence that the use of free nipple graft leads to a decreased chance of recurrence as opposed to the pedicled technique (*p* = 0.005) [20]. Anecdotal report of a successful reduction with modified superior pedicle method has been described [7].

In the treatment of our patient, we adhered to this limited available evidence and favored the breast reduction surgery with free nipple graft technique as the first line treatment option. During the 5-year follow-up period of our patient, there was no recurrence noted. This clinical course of our patient represents a longer follow-up period than most reported studies. This demonstrates the long-term reliability of this technique in this singular case of a JHB. Concomitantly, we hope this could minimize the reporting bias that has often been the subject of scrutiny for many case reports. At this stage, the secondary deformity from our reduction was evident. Secondary revision was offered but it was declined. She remains asymptomatic and satisfied with the aesthetic outcome, albeit the aesthetic result was only acceptable from an objective point of view; that is, symptomatic relief and avoidance of recurrence were achieved.

Medical therapies, mainly hormone modulators, have been attempted in the treatment of JHB. These include tamoxifen, dydrogesterone, medroxyprogesterone (Depo-provera), bromocriptine, and danazol. Tamoxifen is a selective oestrogen receptor modulator (SERM) and is the most commonly used medical therapy in recent literature. The dose of tamoxifen ranged from 10 to 40 mg/day with varying dosing schedule [3, 13]. However, the evidence of its efficacy is variable between reported cases and the long-term safety of its use in adolescent women is unknown. The well-known side effects of tamoxifen include increased risk of endometrial cancer, thromboembolism, hot flashes, and decrease in bone density [1, 3, 13]. Our patient demonstrated a stable disease following the operation and was not commenced on any medical therapy. A comprehensive review of the medical therapies in JHB has been described elsewhere [6, 10, 19] and is outside the scope of this article.

## 4. Conclusion

This present case is unique as it demonstrates the clinical course of this patient at a considerably longer follow-up period than most reported studies. In the treatment of this patient, we adhered to the limited available evidence from the literature. We highlight the long-term reliability of breast reduction with free nipple grafting as the first line surgical option in a young girl with JHB, eliminating the need for repeated surgeries. JHB has only been described sporadically in the literature and this case will further contribute to the knowledge and future studies in establishing the optimal treatment modality for this debilitating condition.
